# Androgenetic alopecia in polycystic ovary syndrome: a cutaneous marker of systemic metabo-inflammatory and endocrine dysfunction

**DOI:** 10.1530/EC-25-0728

**Published:** 2026-05-28

**Authors:** Farzaneh Motafeghi, Marzieh Saei Ghare Naz, Fahimeh Ramezani Tehrani, Samira Behboudi-Gandevani

**Affiliations:** ^1^ Reproductive Endocrinology Research Center, Research Institute for Endocrine Molecular Biology, Research Institute for Endocrine Sciences, Shahid Beheshti University of Medical Sciences, Tehran, Iran; ^2^ Foundation for Research & Education Excellence, Vestavia Hills, Alabama, USA; ^3^ Faculty of Nursing and Health Sciences, NORD University, Bodø, Norway

**Keywords:** polycystic ovary syndrome, androgenetic alopecia, hyperandrogenism, insulin resistance, hair follicle miniaturization, genetic predisposition

## Abstract

Polycystic ovary syndrome (PCOS) is a multifaceted endocrine–metabolic disorder in which androgenetic alopecia (AGA) serves as a prominent clinical marker of profound systemic dysregulation, extending beyond simple hyperandrogenism. This condition imposes a significant psychosocial burden on affected women, yet its complete pathophysiology remains incompletely understood, leading to therapeutic plateaus. This review advances the central hypothesis that alopecia in PCOS results from a synergistic failure of local follicular metabolic signaling and genetic predisposition. This framework posits that the hair follicle in susceptible individuals is a site of intrinsic vulnerability where systemic insults, principally insulin resistance and chronic low-grade inflammation, converge with specific gene polymorphisms. This local pathology both amplifies and is amplified by systemic hyperandrogenism, rendering androgen action a necessary but insufficient component of the complete pathophysiological cascade. This integrated perspective recontextualizes the roles of insulin, inflammatory mediators, and genetic variants as direct effectors of follicular distress, not merely as upstream triggers of androgen excess. We critically synthesize the evidence supporting this model, examining the intricate intersections of systemic hormone bioavailability, local androgen conversion, follicular bioenergetics, and genetic susceptibility. Furthermore, we provide an evidence-based, mechanistically organized framework for the management of PCOS-associated alopecia, evaluating therapeutic modalities based on their targeted action within this complex network. A deeper, systems-level understanding of this interplay is essential for moving beyond current therapeutic limitations and developing personalized management strategies that address the complete pathophysiological axis, ultimately improving both cutaneous and long-term systemic health outcomes for women with PCOS.

## Introduction

Polycystic ovary syndrome (PCOS) is the most common endocrine disorder affecting women of reproductive age, with a global prevalence estimated between 6 and 15%. Once considered primarily a gynecological condition, it is now recognized as a systemic metabolic disruption with profound reproductive, metabolic, and psychological implications (1). Its heterogeneous clinical presentation, including hyperandrogenism, ovulatory dysfunction, and polycystic ovarian morphology, may reflect this multisystemic nature of the syndrome (2, 3).

Within this spectrum, cutaneous manifestations, such as hirsutism, acne, and androgenetic alopecia (AGA), also known as female pattern hair loss (FPHL), serve as important external markers of internal hormonal and metabolic disturbances (4). Affecting 20–35% of women with PCOS, AGA is often distressing, contributing to impaired quality of life and increased anxiety and depression (5).

**Figure 1:**
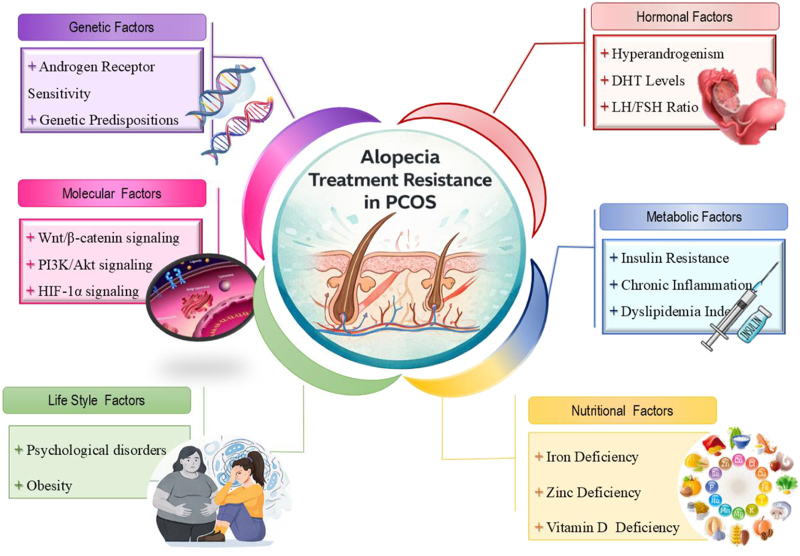
Schematic model for synergistic pathology hypothesis.

The traditional androgen-centric model, which attributes follicular miniaturization to excess dihydrotestosterone (DHT) acting on genetically susceptible follicles (6), fails to adequately explain the condition in women who have normal circulating androgen levels (7). This paradox suggests that local follicular sensitivity, influenced by genetic and metabolic factors, may be as crucial as systemic androgen level (8, 9, 10). This review advances a synergistic pathology hypothesis. We posit that PCOS-associated alopecia arises from systemic metabolic and hormonal dysregulation, encompassing insulin resistance, chronic low-grade inflammation, and hyperandrogenism. These systemic factors amplify intrinsic local follicular vulnerabilities caused by defective metabolic signaling and genetic predisposition. In this model, androgens act as conditional amplifiers rather than the sole drivers of follicular pathology. This integrated framework thereby redefines androgenetic alopecia (AGA) as a cutaneous marker of underlying metabolic dysfunction, providing a novel paradigm to inform phenotype-specific therapies and targeted interventions at the follicular level. This hypothesis critically synthesizes evidence from diverse studies, addressing limitations in the androgen-centric model and proposing a comparative framework for phenotype-based interventions (Fig. 1).

### Alopecia and its types

Alopecia encompasses a spectrum of hair loss disorders with distinct etiologies and clinical presentations (11). Accurate classification is essential, as each type has unique prognostic and therapeutic implications. Alopecia is broadly categorized as non-scarring (non-cicatricial) and scarring (cicatricial). In non-scarring alopecia, the hair follicle structure remains intact, allowing potential regrowth; in scarring alopecia, irreversible destruction of the hair follicle leads to fibrosis (12).

In women with PCOS, the main differential diagnoses for hair loss are three non-scarring types: androgenetic alopecia (AGA), telogen effluvium (TE), and alopecia areata (AA). Distinguishing between these is critical, as their co-existence can complicate the clinical picture and management strategy (13).

Androgenetic alopecia is the most common form of hair loss in PCOS (5). It is a genetically determined condition characterized by a progressive miniaturization of androgen-sensitive hair follicles (14). This process shortens the anagen growth phase and prolongs the telogen resting phase of the hair cycle, producing finer, shorter, and less pigmented hairs (11, 15). Clinically, this manifests as diffuse thinning over the vertex and mid-frontal scalp, often with preserved frontal hairline, commonly forming the Christmas tree or Olsen pattern (7). In PCOS, the systemic hyperandrogenism and metabolic milieu act synergistically to accelerate this process (16).

Telogen effluvium (TE) is a form of diffuse, non-patterned hair shedding triggered by stressors that push hair follicles prematurely into the telogen phase (17). Triggers for hair loss can include physiological factors, such as postpartum hormonal shifts or major surgery, psychological stress, or metabolic conditions, such as nutritional deficiencies or acute illness (18). In PCOS, chronic systemic stress, inherent hormonal fluctuations, and metabolic disturbances can precipitate or exacerbate TE (19, 20). TE often co-exists with AGA, exacerbating or revealing underlying AGA, leading to a more pronounced and acute hair loss presentation that poses diagnostic challenges (4, 21). Alopecia areata is an autoimmune disorder characterized by a T-lymphocyte-mediated attack on the hair follicle bulb, leading to a rapid cessation of hair growth and patchy hair loss (22, 23). Although its pathogenesis differs, AA must be ruled out in PCOS patients, as the syndrome’s chronic low-grade inflammation (24) may heighten immune dysregulation, potentially increasing susceptibility to AA-like presentations (25).

### Androgen action and hair follicle dynamics

The human hair follicle is a complex mini-organ that is regulated by endocrine, paracrine, and autocrine signals, with androgens playing a dual role (26, 27). In regions such as the face and chest, androgens promote terminal hair growth, while in the scalp vertex, they drive follicular miniaturization in AGA due to variations in androgen receptor (AR) density and 5α-reductase activity (28, 29). In AGA, testosterone is converted by 5α-reductase (type 2, predominant in scalp follicles) into DHT, a potent androgen that binds ARs in dermal papilla cells (DPCs) with high affinity (30, 31). This AR-DHT complex translocates to the nucleus to modulate growth factors, such as TGF-β1, DKK1, and IGF-1. This modulation shortens the anagen phase and causes progressive follicle shrinkage, ultimately producing finer and shorter hairs (32, 33).

In PCOS, this androgenic pathway is highly vulnerable, amplified by systemic hyperandrogenemia and local metabolic, inflammatory, and genetic factors (34, 35). Androgens also induce pro-inflammatory cytokines, fostering perifollicular inflammation that exacerbates miniaturization in follicles pre-sensitized by genetic factors, such as HSD3B1 polymorphisms (36, 37). Thus, androgen action in PCOS-associated alopecia is a conditionally pathogenic process, reliant on a hyper-responsive follicular environment rather than circulating androgen levels alone (7, 38).

### Non-androgenic hormone landscape of PCOS and alopecia

Emerging evidence suggests that the pathophysiology of PCOS extends to neuroendocrine dysregulation, specifically involving the gamma-aminobutyric acid (GABA) system. GABAergic dysfunction in the hypothalamus contributes to increased GnRH and LH pulsatility, thereby driving ovarian hyperandrogenism, which fuels follicular miniaturization (39). Furthermore, reduced systemic GABA levels have been correlated with metabolic disturbances, including insulin resistance and dyslipidemia, creating a vicious cycle that exacerbates the metabolic burden on the hair follicle (40).

While closely linked, insulin resistance (IR) and compensatory hyperinsulinemia contribute to alopecia through distinct pathophysiological mechanisms. Systemically, compensatory hyperinsulinemia acts as a potent driver of androgen excess. It synergizes with luteinizing hormone (LH) to stimulate ovarian theca cells, increasing the production of testosterone and androstenedione (41). Concurrently, high insulin levels suppress hepatic synthesis of sex hormone-binding globulin (SHBG), thereby elevating bioavailable testosterone levels that enhance follicular androgen signaling (15, 42). In contrast, insulin resistance directly compromises local follicular function. The hair follicle is a highly proliferative organ that requires significant energy. IR impairs insulin/IGF signaling and alters glucose handling in skin cells. Furthermore, it impairs cutaneous microvascular perfusion, which compromises perifollicular nutrient delivery (43, 44, 45). This metabolic blockade limits oxygen and nutrient delivery, creating a local energy deficit that shortens the anagen phase and promotes miniaturization, independent of circulating androgen levels (46).

Elevated LH, driven by altered GnRH signaling, further fuels ovarian androgen production, while reduced aromatase activity (CYP19 gene) in ovarian follicles shifts the hormonal balance toward androgen dominance, impairing follicle development and worsening hyperandrogenic symptoms, such as alopecia (47, 48). Thus, in PCOS, insulin, LH, and aromatase form an interconnected hormonal network that amplifies androgen action and metabolic stress, driving progressive hair loss through a hyper-responsive follicular environment (48).

### Genetics landscape of PCOS and alopecia

The clinical expression of AGA in women with PCOS is shaped by genetic predisposition, which determines hair follicle sensitivity to hormonal and metabolic stressors (49). As a polygenic condition, AGA is influenced by variants in genes such as HSD3B1, CYP17, and CYP19A1, which modulate androgen synthesis, metabolism, and receptor signaling. These genetic differences explain why women with comparable hormonal profiles may exhibit markedly different degrees of hair loss (50). These variants act at distinct points in the androgenic pathway, amplifying follicular sensitivity as outlined below:

- **Local androgen metabolism and sensitivity:** Evidence shows that a variant in the HSD3B1 (rs1047303, the 1245C) allele enhances 3β-hydroxysteroid dehydrogenase activity, leading to increased local synthesis of testosterone and DHT in the skin (37). This variant is strongly associated with female pattern hair loss in PCOS, particularly in overweight or obese women, highlighting the role of follicular hypersensitivity over systemic androgen levels (37).
- **Systemic androgen synthesis:** Genetic variations can also modulate the systemic production of androgens. Polymorphisms in the CYP17 gene, which encodes a key enzyme in steroidogenesis, have been linked to increased enzyme activity, resulting in higher circulating androgen levels, and an increased risk of alopecia (51). However, this systemic androgen excess complements local follicular hypersensitivity, driving the hyperandrogenic phenotype in PCOS.
- **Androgen-estrogen balance:** The CYP19A1 gene variant (rs2414096, G allele) encodes aromatase, which converts androgens to estrogens. It reduces aromatase activity, shifting the hormonal balance toward androgen dominance and increasing susceptibility to PCOS and AGA (48). While AR gene polymorphisms are well-established in male-pattern baldness, their role in FPHL remains less clear, suggesting sex-specific genetic influences (52).

However, it should be noted that these genetic associations lack consistent replication across diverse global populations. This lack of data makes their current application for clinical risk prediction or personalized treatment uncertain.

### Metabolic landscape of PCOS and alopecia

Metabolic dysfunction is a recognized hallmark of PCOS. Abnormalities such as insulin resistance, dyslipidemia, and inflammatory dysregulation are strongly associated with female pattern hair loss in these patients. This suggests that these factors adversely affect the follicular microenvironment directly, extending beyond their contribution to hyperandrogenism, as detailed below (37, 53, 54):

- **Insulin resistance as a direct follicular toxin:** Insulin resistance and compensatory hyperinsulinemia in PCOS contribute to alopecia beyond its systemic effects on ovarian androgen production and SHBG suppression. At the tissue level, IR reflects impaired insulin signaling within target cells, including keratinocytes and dermal papilla cells, independent of circulating insulin concentrations. The hair follicle is a highly proliferative mini-organ with substantial energy demands during the anagen phase, rendering it particularly vulnerable to intracellular defects in glucose uptake and utilization. Impaired insulin signaling reduces glucose transport, disrupts mitochondrial function, and compromises keratinocyte proliferation, leading to energy deficiency, premature anagen termination, and follicular miniaturization (55, 56). Local insulin resistance is further associated with endothelial dysfunction, reduced microvascular perfusion, and increased oxidative stress. Collectively, these factors impair oxygen and nutrient delivery to the follicular unit. They also amplify inflammatory pathways that potentiate androgen-mediated hair loss (55). Hyperinsulinemia represents a systemic compensatory response to IR and contributes to AGA primarily through endocrine and paracrine effects. Elevated insulin levels suppress hepatic sex hormone-binding globulin synthesis and enhance ovarian and adrenal androgen production, thereby increasing free androgen bioavailability at the follicular level (57). In addition, insulin directly activates insulin-like growth factor-1 (IGF-1) signaling pathways within the hair follicle, which may sensitize androgen receptors and exacerbate follicular miniaturization. Mendelian randomization studies demonstrate a causal association between genetically determined fasting insulin levels and increased AGA risk, independent of other metabolic traits, supporting a direct pathogenic role for hyperinsulinemia beyond generalized metabolic dysfunction (58, 59).
- **Dyslipidemia and lipotoxicity:** Dyslipidemia in PCOS commonly features low HDL-C and elevated triglycerides. In insulin-resistant states, elevated circulating free fatty acids may promote ectopic lipid stress in peripheral tissues. Free-fatty-acid-induced lipotoxicity is mechanistically linked to ER stress/UPR activation, mitochondrial dysfunction, and ceramide accumulation – pathways that could plausibly compromise follicular cell function (60, 61, 62). This lipotoxic stress disrupts membrane integrity and key signaling pathways essential for anagen maintenance. Notably, it impairs peroxisome proliferator-activated receptor-γ (PPAR-γ) signaling, which regulates lipid homeostasis and anti-inflammatory responses in dermal papilla cells. It also downregulates Wnt/β-catenin signaling, which is critical for follicular proliferation and stem cell activation. Consequently, these disruptions promote oxidative damage, local inflammation, premature catagen entry, and progressive follicular shrinkage, establishing lipid imbalance and lipotoxicity as an independent metabolic axis amplifying follicular injury in PCOS-associated alopecia (63, 64, 65, 66, 67).
- **Chronic inflammation and adipose dysfunction in PCOS-associated alopecia:** Low-grade inflammatory dysregulation is frequently reported in PCOS and may link metabolic dysfunction to adverse effects on hair follicle homeostasis. Adipose tissue dysfunction, present even in some lean individuals and typically amplified with obesity, can be associated with higher pro-inflammatory mediators (such as leptin and TNF-α) and lower adiponectin. These signals may promotec an oxidative-stress-prone perifollicular milieu, contributing to impaired follicular regeneration and pro-apoptotic signaling (68, 69, 70, 71, 72). Obesity, particularly increased visceral adiposity, can exacerbate insulin resistance, dyslipidemia, and inflammatory dysregulation in PCOS and is associated with greater hyperandrogenism. Accordingly, AGA/FPHL in PCOS may function as a cutaneous marker of broader metabolic dysfunction, supporting interventions that address both metabolic and inflammatory factors (53, 68, 73).

### Molecular landscape of PCOS and alopecia

At the cellular and molecular level, PCOS-associated alopecia reflects dysregulated signaling pathways within the hair follicle that integrate androgenic, metabolic, and inflammatory cues, leading to a shortened anagen phase and progressive reduction in follicular size (74). Dermal papilla cells (DPCs), the key mesenchymal compartment orchestrating follicular cycling, exhibit altered gene expression profiles in response to hyperandrogenic and insulin-resistant microenvironments. AR activation in DPCs upregulates inhibitory factors, such as TGF-β1 and dickkopf-1 (DKK-1), which suppress Wnt/β-catenin signaling, a critical pathway for anagen prolongation and follicular stem cell activation (33). Hyperinsulinemia and insulin activate PI3K/Akt signaling in skin cells, supporting proliferative and survival responses. However, dysregulated insulin/IGF signaling and a reduction in anagen-maintaining factors (such as IGF-1) can trigger premature catagen, a process further exacerbated by oxidative stress (75, 76, 77). Chronic low-grade inflammation further exacerbates molecular distress via nuclear factor-κB (NF-κB) activation, elevating pro-inflammatory cytokines, including TNF-α and IL-6, which induce apoptosis in matrix keratinocytes and impair mitochondrial bioenergetics in DPCs (78). Lipid dysregulation contributes through PPAR-γ suppression, disrupting fatty acid oxidation and amplifying lipotoxic effects on follicular energy homeostasis (79). It has been shown that pathways involving extracellular matrix remodeling, apoptosis, and hypoxia-inducible factor-1α (HIF-1α) signaling overlap with molecular signatures observed in PCOS adipose and ovarian tissues (35). These converging molecular perturbations suggest that the hair follicle actively senses systemic metabolic disruptions. Genetic variants further modulate this pathway sensitivity, leaving follicles vulnerable to synergistic failure even without severe hyperandrogenemia.

## Clinical presentation and diagnosis of alopecia in PCOS

AGA in PCOS typically presents as female pattern hair loss, characterized by diffuse thinning at the vertex and mid-frontal scalp, with the frontal hairline preserved (11). Common patterns include the Ludwig type (centrifugal widening of the central part) and the Olsen type (Christmas tree-shaped thinning toward the front) (80). The Ludwig scale grades severity: Stage I (early thinning), Stage II (visible scalp), and Stage III (sparse crown hair) (81). Prevalence varies widely (3.2–76.9%) due to diagnostic and population differences, with a 2023 study noting 9.4% of Ludwig-type FPHL cases having PCOS (82, 83). Notably, a substantial proportion of women with moderate-to-severe FPHL lack other clinical signs of hyperandrogenism, such as hirsutism, and exhibit circulating androgen concentrations within standard laboratory reference ranges (84). However, androgen levels in this population may cluster toward the upper end of the normal range. Biologically relevant androgen activity is often more accurately reflected by composite indices, such as the free androgen index, even if formal diagnostic thresholds for biochemical hyperandrogenism are not met. These findings support the concept that local follicular androgen sensitivity, modulated by genetic predisposition, altered sex hormone-binding globulin levels and metabolic factors, and plays a central role in the pathogenesis of FPHL (84).

In addition, alopecia in PCOS imposes a significant psychosocial burden, extending beyond physical symptoms to impair mental health (10, 11).

In women, the loss of healthy hair, especially at a younger age, is linked to diminished self-esteem and a decreased sense of attractiveness and femininity, often contributing to lower confidence levels (85). This can result in feelings of frustration, sadness, and a general decline in overall well-being and impaired quality of life (86). Additionally, hair loss may heighten stress and social anxiety due to concerns about how others perceive or react to their appearance (87). Over time, these challenges can lead to reduced participation in social activities and gradual social withdrawal (88).

Diagnosing AGA in PCOS requires integrating clinical and laboratory assessments to confirm PCOS-related drivers and rule out other causes (89). A detailed history should cover hair loss onset, pattern and progression, menstrual irregularities, and family history of alopecia or PCOS. Physical examination, using the Ludwig or Sinclair scales, evaluates hair loss severity and identifies hyperandrogenic signs (hirsutism, acne, and acanthosis nigricans) (7). Laboratory tests include serum total and free testosterone, androstenedione, and DHEAS to assess hyperandrogenism. Evaluating LH/FSH ratios may provide supportive evidence for PCOS, although it is not required for a formal diagnosis. Metabolic evaluation, including fasting glucose, insulin, HOMA-IR, and lipid profile, detects insulin resistance and dyslipidemia. Tests for thyroid function (TSH), ferritin, and prolactin exclude alternative causes, such as thyroid dysfunction or iron deficiency. Scalp biopsy is rarely needed but may differentiate AGA from scarring alopecia in unclear cases (89, 90).

## Management of alopecia in PCOS

The management of PCOS-associated alopecia is challenging and requires an integrated multi-targeted approach that addresses the interplay of androgen excess, insulin resistance, and inflammation. The following framework summarizes therapeutic options according to their primary mechanism of action and the strength of supporting evidence. However, although multiple therapeutic approaches for androgenetic alopecia in PCOS have been proposed, the supporting evidence remains limited. Most available studies are characterized by small sample sizes, heterogeneous treatment protocols, and short follow-up durations, which substantially restrict the robustness, reproducibility, and generalizability of their findings. Consequently, current data are insufficient to support definitive, evidence-based clinical recommendations. Well-designed, adequately powered randomized controlled trials with standardized outcome measures are, therefore, warranted.

**First-line therapy**: Lifestyle and metabolic management. In accordance with the 2023 International Evidence-based Guidelines, lifestyle intervention, encompassing diet, exercise, and behavioral strategies, is the primary treatment for all women with PCOS, regardless of phenotype (89). While specific clinical trials evaluating the isolated effect of lifestyle modification on hair regrowth are limited, its impact on the underlying drivers of alopecia is well established. Weight loss and physical activity significantly reduce IR and compensatory hyperinsulinemia, which in turn lowers ovarian androgen production and increases sex hormone-binding globulin (SHBG) levels. By mitigating systemic metabolic dysfunction and chronic low-grade inflammation, lifestyle modifications reduce the synergistic load on the hair follicle. This restoration of metabolic homeostasis is critical; without it, follicles remain in a state of energy deprivation and oxidative stress, rendering pharmacological treatments, such as minoxidil or anti-androgens less effective (89).

### Direct follicular growth stimulants

These agents act directly on the hair follicle to stimulate growth, largely independent of systemic hormonal effects.

- **Topical minoxidil (2% or 5%):** The only FDA-approved treatment for FPHL, topical minoxidil is the first-line therapy. It acts by prolonging the anagen phase and enhancing perifollicular blood flow (91). Although large-scale randomized trials exclusively in PCOS cohorts are limited, evidence from broader FPHL populations demonstrates its efficacy, with the 5% foam showing superiority over the 2% solution in increasing hair density (91). However, response in PCOS may vary by phenotype; a comparative trial indicated that while minoxidil is effective, women with marked hyperandrogenism may show a superior response to anti-androgens (such as cyproterone acetate) compared to minoxidil monotherapy (92). Thus, for many women with PCOS, minoxidil acts best as a foundational treatment alongside systemic androgen modulation. Approximately 60% of patients experience partial regrowth, although continuous application is required to maintain results. Adverse effects, such as scalp irritation and facial hypertrichosis, are generally mild and manageable.
- **Low-dose oral minoxidil:** It is a promising off-label alternative (0.25–2.5 mg daily), particularly for patients intolerant to topical therapy. Evidence suggests its efficacy is comparable to topical minoxidil. However, patients must be carefully monitored for systemic side effects, such as generalized hypertrichosis, fluid retention, and tachycardia (93).

### Androgen pathway modulators

These agents target the hormonal driver of AGA and are particularly suited for women with clinical or biochemical hyperandrogenism.

- **Systemic anti-androgens (spironolactone):** A potassium-sparing diuretic and androgen antagonist, spironolactone remains a cornerstone of off-label therapy for FPHL in PCOS. Original clinical studies have demonstrated significant clinical improvement and a reduction in hair loss progression in female patients treated with oral anti-androgens. Effective doses typically range from 100–200 mg/day (94, 95). Menstrual irregularities, breast tenderness, and fatigue are the most common side effects; contraception is essential due to teratogenic risk (96, 97).
- **5α-reductase inhibitors (finasteride and dutasteride):** These agents block the conversion of testosterone to DHT, the key androgen involved in the reduction of follicular size (98). Dutasteride inhibits both type I and II isoenzymes, making it more potent than finasteride, which primarily targets type II (99). Evidence supports efficacy at higher doses or in hyperandrogenic women, although use in premenopausal patients remains off-label and requires reliable contraception due to teratogenicity (98).
- **Combined oral contraceptives (OCPs):** OCPs containing estrogen and a low- or anti-androgenic progestin suppress ovarian androgen production and increase hepatic SHBG synthesis, thereby reducing free testosterone levels. While their direct effect on hair regrowth is modest, OCPs are essential for hormonal regulation and for providing contraception during anti-androgen therapy (4).
- **Cyproterone acetate (CPA):** A potent progestin and androgen receptor antagonist, CPA (often combined with ethinyl estradiol) is widely used outside the United States (100). Clinical trials support improvement in over 80% of PCOS patients after 6 months of therapy. Potential side effects include weight gain, mood changes, and an increased risk of venous thromboembolism (101).

### Adjuvant and emerging therapies

These modalities serve as adjuncts to pharmacologic therapy or alternatives for patients who cannot tolerate systemic agents.

- **Low-level laser therapy (LLLT):** LLLT stimulates follicular activity through photobiomodulation and improved mitochondrial function. The evidence level is low to moderate, with a meta-analysis showing a significant increase in hair density. It is a safe and well-tolerated option, but efficacy is modest and requires consistent, long-term use (102).
- **Mesotherapy:** Also known as intradermotherapy, this technique involves intradermal injection of diluted active agents, such as minoxidil, finasteride, or growth factors, to enhance local efficacy and minimize systemic exposure (9, 103). Despite growing use, lack of standardized protocols, discomfort from repeated injections, and variable results limit its reliability.
- **Platelet-rich plasma (PRP) therapy:** PRP delivers autologous growth factors, including platelet-derived growth factor (PDGF), transforming growth factor beta (TGF-β), vascular endothelial growth factor (VEGF), epidermal growth factor (EGF), and IGF-1, that stimulate follicular stem cells and angiogenesis (104, 105). Randomized trials and meta-analyses demonstrate significant improvements in hair density and thickness, although results are constrained by small sample sizes and heterogeneity in preparation methods. Furthermore, trials specifically evaluating PRP in women with PCOS are limited. Given the unique inflammatory and metabolic milieu of PCOS, further research is needed to confirm whether efficacy in this subgroup matches that of the general population, and standardized protocols are essential to optimize outcomes (106).

### Foundational metabolic and nutritional support

Addressing the underlying metabolic dysfunction is essential for long-term success and improved follicular resilience.

- **Metformin and lifestyle intervention:** Lifestyle modification and insulin-sensitizing agents, such as metformin, are fundamental to managing insulin-resistant PCOS. Although metformin has limited direct effect on hair regrowth, it improves insulin sensitivity, reduces hyperinsulinemia, lowers androgen levels, and reduces follicular metabolic stress, thereby improving the efficacy of other targeted hair therapies (4).
- **Nutritional supplements:** Correcting documented deficiencies in iron, vitamin D, and zinc is important for overall hair health, although evidence for supplementation in non-deficient individuals remains limited (107, 108).

## Factors associated with suboptimal therapeutic response

Despite the range of available therapeutic modalities, a considerable proportion of women with PCOS-associated alopecia experience limited or unsatisfactory responses to treatment. This so-called treatment resistance often reflects a mechanistic mismatch, where interventions target only part of the underlying pathophysiology. Understanding the factors that contribute to suboptimal response is essential for realistic counseling and more precise, individualized management (91, 109). Figure 1 summarizes the major factors influencing treatment response in PCOS-associated alopecia, focusing on the interplay between hormonal, metabolic, genetic, nutritional, and lifestyle determinants.

Persistent metabolic dysfunction is a primary determinant of poor response. A therapeutic strategy focused solely on blocking androgen action with agents such as spironolactone or finasteride may fail when severe insulin resistance and inflammation remain unaddressed (110). In such cases, hair follicles remain metabolically stressed and unable to respond adequately to growth stimuli. Restoring metabolic homeostasis is, therefore, fundamental to achieving meaningful regrowth (11, 15).

Genetic variability also modulates therapeutic efficacy. Polymorphisms in the androgen receptor gene may alter the receptor expression or affinity, reducing responsiveness to antagonists, while variations in 5α-reductase activity can affect inhibition efficiency (111, 112).

The duration and severity of alopecia at the time of intervention strongly predict hair regrowth outcomes. In advanced follicular size reduction, significant regression of terminal follicles and replacement with fibrous tissue limit regrowth potential, emphasizing the need for early intervention (113).

The presence of undiagnosed comorbidities can confound the clinical picture and inhibit therapeutic response. Overlapping telogen effluvium, due to metabolic or psychological stress, iron deficiency, or thyroid dysfunction, can cause diffuse shedding that masks the progression of AGA and must be identified and corrected to optimize results (107, 108).

**Figure 2:**
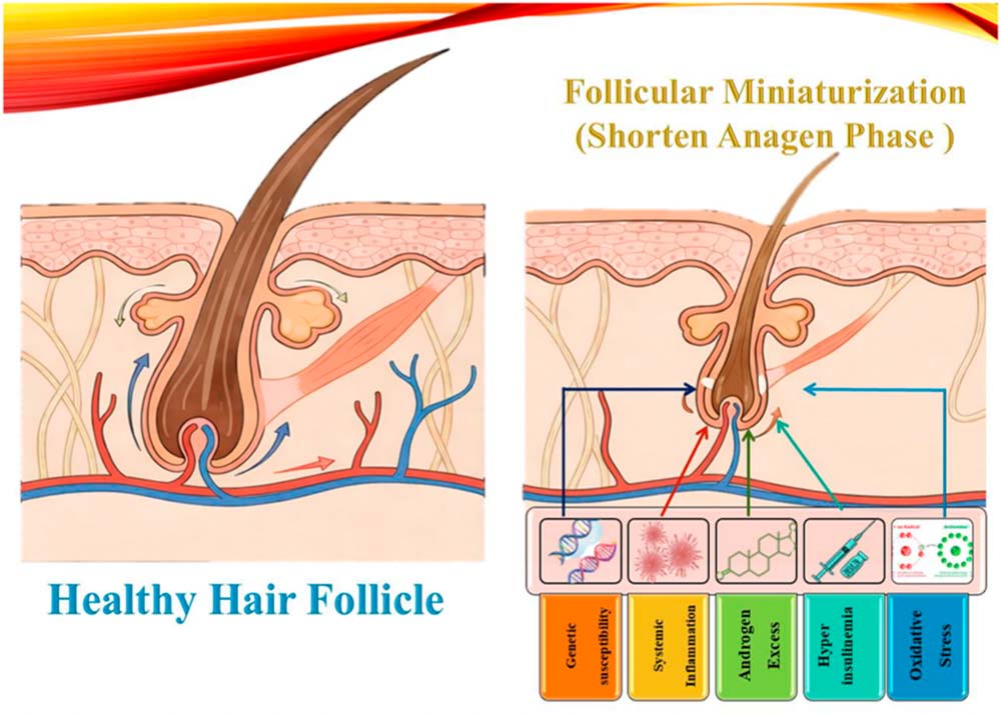
Factors associated with suboptimal therapeutic response.

Finally, patient-related factors, including adherence to treatment, remain a critical yet often overlooked factor. Most therapies require sustained, consistent use over months to yield visible improvement. Premature discontinuation, common with agents such as minoxidil or anti-androgens, can lead to perceived ineffectiveness when, in reality, the therapeutic duration was insufficient (86, 95). Figure 2 presents Factors associated with suboptimal therapeutic response.

## Research gaps and future directions

PCOS-related alopecia remains an underexplored aspect of the syndrome, challenged by diagnostic limitations, therapeutic variability, and fragmented research approaches. Future advancement depends on characterizing the follicular microenvironment and adopting precision-based, phenotype-oriented clinical study designs.

A key diagnostic gap lies in the reliance on systemic biomarkers that inadequately represent local follicular pathology. Serum androgen levels often remain within normal limits even in women with advanced FPHL, making them an unreliable indicator for scalp-specific androgenic or metabolic activity. There is a critical need for novel, localized biomarkers that capture the biochemical milieu of the follicle. Techniques such as minimally invasive sampling of plucked hairs or scalp micro-biopsies could be highly beneficial. They allow for the assessment of key genes, including AR and SRD5A2 (5α-reductase type 2), and inflammatory metabolic regulators, providing a more accurate reflection of the local follicular microenvironment.

Therapeutically, evidence remains constrained by the limited number of high-quality, comparative clinical trials within well-characterized PCOS cohorts. Most pharmacological treatments are used off-label, with uncertainties regarding optimal dosing, long-term safety, and differential efficacy among PCOS phenotypes. Furthermore, most existing treatments largely stabilize or slow hair loss rather than reverse follicular size reduction, highlighting a need for therapies with true regenerative strategies capable of restoring follicular structure and function.

Based on the synergistic failure hypothesis advanced in this review, future research should prioritize the following directions:

- **Defining the follicular microenvironment:** High-throughput transcriptomic and proteomic analyses of scalp tissue from women with PCOS-associated alopecia could elucidate the molecular signatures of metabolic stress, inflammation, and dysregulated androgen signaling within follicular compartments. Such data may reveal novel, druggable pathways for restoring local follicular homeostasis (114, 115).
- **Phenotype-stratified clinical trials:** Future therapeutic trials should adopt a precision-based design, stratifying participants by dominant PCOS phenotype to clarify differential treatment responses. Comparative studies, such as spironolactone versus metformin plus minoxidil in insulin-resistant cohorts, could refine phenotype-specific therapeutic algorithms.
- **Investigating direct metabolic and inflammatory pathways:** Basic research is needed to clarify how hyperinsulinemia, dyslipidemia, and pro-inflammatory cytokines directly impact human dermal papilla cell function, proliferation, and gene expression. Understanding these pathways could lead to the development of novel topical or systemic therapies that specifically target follicular metabolic failure.
- **Longitudinal and psychological studies:** More research is required to address the psychological burden of alopecia in this population and to develop integrated care models that encompass both the dermatologic and mental health aspects of the condition.

## Psychosocial burden and quality of life of female alopecia

Alopecia in women with PCOS is not merely a cosmetic concern but a significant source of psychological distress, often exceeding that of other cutaneous manifestations, such as acne or hirsutism. Emerging evidence indicates that hair loss profoundly impacts self-esteem, perception of femininity, and social interaction (116). The synergistic failure of hair growth mirrors a perceived loss of control over one’s body, contributing to higher rates of anxiety, depression, and dysmorphic body concerns in this population (117, 118). Consequently, the success of therapeutic interventions should not be measured solely by hair density counts, but by the restoration of psychosocial well-being. The psychological distress associated with alopecia in PCOS may be biologically underpinned by altered neurotransmitter profiles. Studies indicate that GABA dysfunction is strongly associated with higher rates of depression and anxiety in women with PCOS, suggesting that the psychosocial burden of alopecia is not merely reactive but may share a common neurobiological etiology with the syndrome itself (40).

## Conclusion

Androgenic alopecia in the context of PCOS is far more than a cosmetic concern; it is a clinical manifestation of a deep-seated and complex interplay between hormonal, metabolic, and genetic factors. The traditional androgen-centric model, while foundational, is insufficient to explain the clinical heterogeneity and therapeutic challenges associated with the condition. This review has synthesized the available evidence to propose a more integrated conceptual framework: PCOS-associated alopecia is a condition of synergistic pathology, where a genetically predisposed hair follicle, already compromised by local metabolic stress and inflammation, becomes hyper-responsive to androgenic stimuli.

This model successfully accounts for the clinical paradox of severe alopecia in the presence of normal systemic androgen levels and provides a mechanistic basis for the variable responses observed with conventional therapies. It reframes the hair follicle not as a passive target but as an active participant in its own pathology, a micro-organ whose fate is determined by the convergence of systemic insults and local vulnerabilities.

The clinical implications of this perspective are profound. The management of alopecia in women with PCOS must evolve from a purely anti-androgenic focus to a holistic, systems-based approach. Effective, long-term management requires the concurrent and aggressive treatment of the underlying metabolic dysfunction, particularly insulin resistance and the associated chronic inflammatory state. By addressing these foundational drivers, clinicians can not only improve the patient’s overall systemic health and reduce long-term cardiometabolic risk but also restore a state of follicular homeostasis that may enhance responsiveness to direct hair growth therapies. Ultimately, alopecia should be viewed by clinicians as a key external indicator for diligent metabolic management, offering an opportunity to improve both the distressing cutaneous symptoms and the lifelong health trajectory of women affected by polycystic ovary syndrome. Addressing these metabolic drivers is critical not only for hair regrowth but for long-term systemic health. While recent Mendelian randomization studies suggest no direct causal genetic link between PCOS and neurodegenerative conditions, such as Alzheimer’s disease, the shared burden of chronic inflammation and insulin resistance highlights the importance of aggressive metabolic management to improve lifelong health trajectories beyond cutaneous manifestations (119).

This novel framework shifts clinical practice toward integrated, precision-based care and directs research toward regenerative therapies, offering a transformative approach to PCOS-associated alopecia. However, it should be noted that the evidence base informing this framework is heterogeneous, encompassing diverse study designs, diagnostic definitions, and outcome measures, which limits direct comparison across studies and constrains the generalizability of individual findings. In addition, some proposed mechanisms rely on preclinical evidence and limited human studies, restricting translational confidence. Furthermore, many studies included in this review focused on specific ethnicities. Given the known ethnic variations in genetic risks, androgen pathways, and hair loss patterns, these findings should be applied cautiously across diverse global populations.

## Declaration of interest

The authors declare that this study was conducted in the absence of any commercial or financial relationships that could be construed as potential conflicts of interest.

## Funding

This study was funded by the Research Institute for Endocrine Sciences, Shahid Beheshti University of Medical Sciences, Tehran, Iran (Grant number: 43017968-IR.SBMU.ENDOCRINE.REC.1404.140). Nord University covered the article processing charge.

## Author contribution statement

FM, MSGN, SB-G, and FRT wrote and reviewed the manuscript.

## AI disclosure

The authors used Copilot and Perplexity for grammatical refinement and text summarization to enhance readability. All AI-assisted content was reviewed and verified by the authors, who take full responsibility for the manuscript’s content and accuracy.
